# A Novel Friction Compensation Method for Machine Tool Drive Systems in Insufficient Lubrication

**DOI:** 10.3390/s24154820

**Published:** 2024-07-25

**Authors:** Yanliang Sheng, Guofeng Wang, Lingling Sang, Decai Li

**Affiliations:** Tianjin Key Laboratory of Equipment Design and Manufacturing Technology, Tianjin University, Tianjin 300072, China; shengyanliang@tju.edu.cn (Y.S.); llsanglls@163.com (L.S.); decaili98@163.com (D.L.)

**Keywords:** nonlinear friction model, periodic harmonic component, friction lag, friction feedforward compensation

## Abstract

Friction is the dominant factor restricting tracking accuracy and machining surface quality in mechanical systems such as machine tool feed-drive. Hence, friction modeling and compensation is an important method in accurate tracking control of CNC machine tools used for welding, 3D printing, and milling, etc. Many static and dynamic friction models have been proposed to compensate for frictional effects to reduce the tracking error in the desired trajectory and to improve the surface quality. However, most of them focus on the friction characteristics of the pre-sliding zone and low-speed sliding regions. These models do not fully describe friction in the case of insufficient lubrication or high acceleration and deceleration in machine tool systems. This paper presents a new nonlinear friction model that includes the typical Coulomb-Viscous friction, a nonlinear periodic harmonic friction term for describing the lead screw property in insufficient lubrication, and a functional component of acceleration for describing the friction lag caused by the acceleration and deceleration of the system. Experiments were conducted to compare the friction compensation performance between the proposed and the conventional friction models. Experimental results indicate that the root mean square and maximum absolute tracking error can be significantly reduced after applying the proposed friction model.

## 1. Introduction

As technology continues to advance, there is a growing demand for higher-performance servo systems in academia and industry. However, the presence of friction significantly constrains the achievable performance, necessitating mitigation measures. Friction compensation has become the key technology of high-performance servo systems, which plays a critical role in improving machining accuracy, and product quality and prolonging service lifetime [1,2]. However, the diversity in the structure of feed drives and the coupling between them lead to highly nonlinear friction behavior [3], thereby rendering the implementation of friction compensation control challenging.

Friction compensation methods can be generally divided into model-free methods and model-based methods [4]. Model-free methods typically treat friction as part of a lumped disturbance, utilizing robust control and adaptive control techniques to mitigate its effects [5,6]. A backstepping motion controller with a disturbance observer was proposed for nonlinear mechanical systems [7]. To reduce the effect of disturbances and parameter variations, a discontinuous adaptive robust controller was constructed for the control of linear motors [8]. Additionally, neural network-based intelligent control algorithms consider friction as a special disturbance that varies with feed velocity, rather than establishing a physical model of friction characteristics [9]. Model-free methods can improve the motion accuracy of drive systems. However, limited by the bandwidth of the servo controller, tracking errors caused by nonlinear friction, which significantly changes near zero speed, still cannot be completely eliminated.

On the other hand, model-based methods leverage the understanding of friction behavior to apply additional driving torque, offsetting the friction disturbance in the actual system [10,11]. This method can effectively eliminate the friction dead zone at velocity reversal, but its performance is highly dependent on the accuracy of the friction model [12,13,14]. Therefore, establishing an appropriate and accurate friction model is the most critical step in model-based methods.

Numerous simple and advanced friction models have been reported to describe friction [15]. Most existing model-based friction compensators use conventional static friction models that describe the static map between velocity and friction torque [16]. Márton et al. [17] deal with friction-induced nonlinearities by linearizing the Stribeck friction model. The conventional static friction model does not describe friction in the pre-sliding regime and is insufficient to represent the friction behavior at very low velocities. Dynamic friction models have been proposed to compensate for these shortcomings. The LuGre friction model is widely applied owing to its simplicity and relatively good performance [18]. De Wit, C. et al. [19] utilized the LuGre friction model for an adaptive friction compensation on a servo motor system. Recently, Al-Bender et al. [20] developed the so-called generalized Maxwell-slip (GMS) friction model. Jamaludin et al. [21] used a dynamic friction model (GMS friction model) for friction compensation techniques and evaluated experimentally on a linear-drive XY table. Park et al. [22] introduced a Sigmoid function friction model for the pre-sliding region and a hybrid friction model correlated with the operating velocity of the sliding region. Yang et al. [23] proposed a hybrid friction model composed of an asymmetric static friction model under pre-sliding conditions and a Tustin model determined based on the critical velocity under sliding conditions. The combined friction model demonstrated superior compensation results compared to the static friction model, as confirmed by simulation experiments. However, the dynamic friction model provides better performance than the conventional static friction model only at a low-velocity regime [24,25]. Furthermore, these models cannot fully describe and compensate for friction in machine tool systems in the case of insufficient lubrication or high acceleration and deceleration motion. This implies that friction is not only correlated with the feed velocity but also significantly influenced by the actual displacement and acceleration of the drive system.

This paper extends the traditional Stribeck friction model by considering nonlinear torque ripple disturbance caused by the eccentricity of the ball screw and nut under conditions of insufficient lubrication, as well as friction lag disturbance during high acceleration processes. A novel extended Stribeck friction model, formulated as a function of feed displacement, velocity, and acceleration, is proposed to accurately describe friction behavior in drive systems. In addition, the parameter identification and friction feedforward compensation strategies are introduced, and the experimental results are discussed to demonstrate the performance of the proposed model.

The rest of this paper is organized as follows. In Section 2, the conventional friction model and proposed friction model are discussed. In Section 3, the identification of friction models is presented. The experimental setup and dynamics models for feed drive systems are presented as well. The experimental results of friction compensation are discussed in Section 4 and conclusions are drawn in Section 5.

## 2. Conventional Friction Model and Proposed Friction Model

### 2.1. Conventional Friction Model

**Figure 1:**
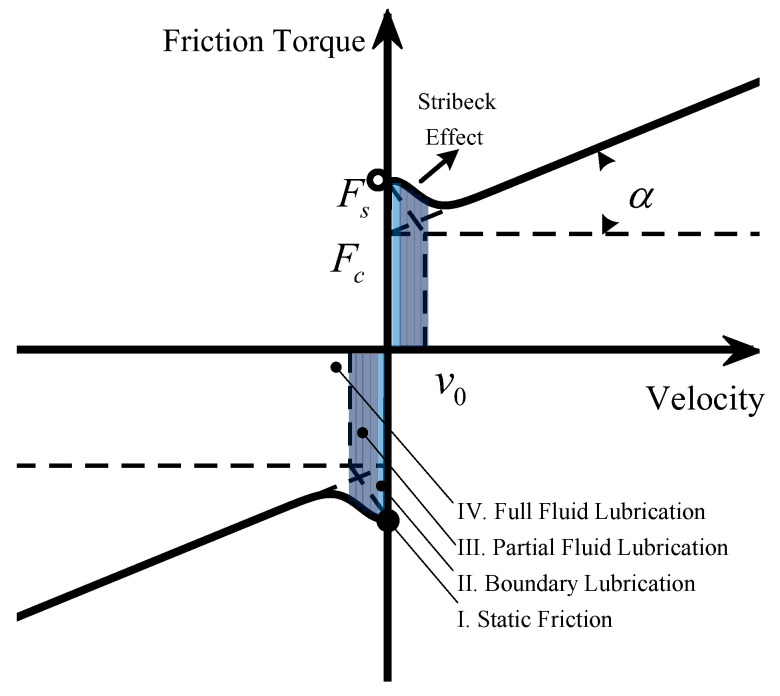
Coulomb-Viscous-Stribeck friction model.

The conventional friction compensation method [19] is based on the typical characteristics of lubricated metallic surfaces, which are described by the Stribeck curve, as shown in Figure 1. There are four regimes of lubrication in a system with grease or oil. With the increase in velocity, it is followed by static friction, boundary lubrication, partial fluid lubrication, and full fluid lubrication. With increasing velocity in the regime of partial fluid lubrication, the friction between two surfaces decreases, which is called the Stribeck effect.

The mathematical equation of the Coulomb–Viscous–Stribeck friction model can be given as

(1) $$T_{f}\left(v\right)=\left[T_{c}+\left(T_{s}-T_{c}\right)e^{-{\left(vv_{0}^{-1}\right)}^{\delta }}\right]sgn\left(v\right)+\alpha v,$$

where $T_{f}$, *v*, $T_{c}$, $T_{s}$, and α are scalar and represent the friction torque, motion velocity, Coulomb torque, static friction, and viscous friction coefficients, respectively. The Stribeck friction model parameters are the Stribeck velocity $v_{0}$ and shape coefficient δ.

### 2.2. Proposed Friction Model

Although the friction model in Equation (1) has been widely used, it considers only nominal linear frictions (e.g., Coulomb, static, and viscous frictions) [19] and considers that friction is only related to the speed of the feed drive system. Especially when the screw lubrication is insufficient or the system starts and stops with high acceleration and deceleration, the above conventional model cannot fully describe the friction characteristics of the system. In this paper, we present a new nonlinear friction model that includes the typical Coulomb-viscous friction, a nonlinear periodic harmonic term that mainly comes from the eccentricity between a lead screw and a nut and a friction lag component caused by the acceleration. Finally, the Stribeck friction model is extended to be a function of acceleration, velocity, and position to fully describe the friction behavior of the feed drive systems.

**Figure 2:**
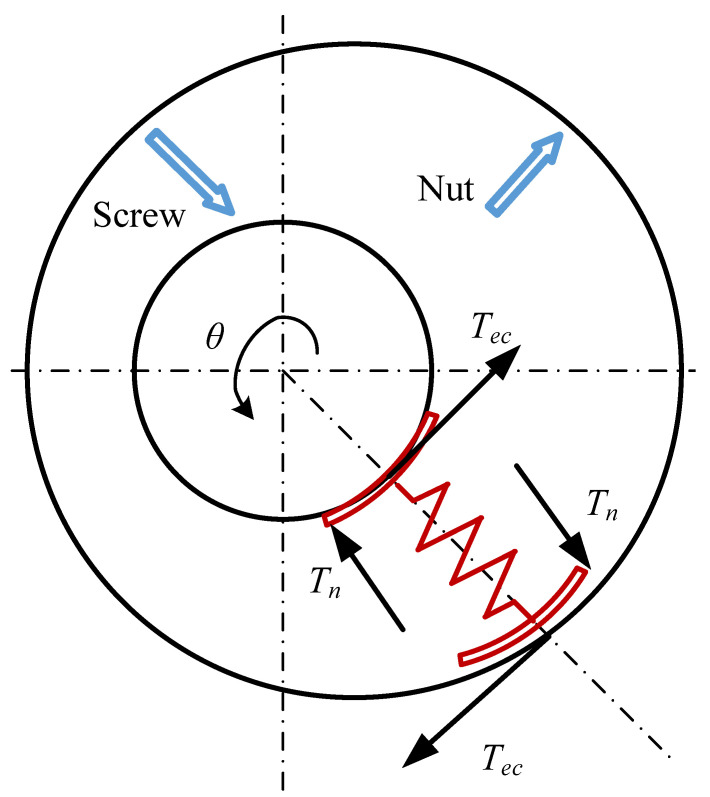
Centrifugal Friction Caused by Ball Screw and Nut Eccentricity.

The mechanism of non-linear friction caused by the eccentricity of the ball screw and nut is shown in Figure 2. In a precise machine tool system, due to the lack of lubrication, the wear between the lead screw and the nut results in an infinitesimal gap causing the friction value to be indeterminate. Based on this assumption, a spring-like model can be used to describe the friction behavior within the screw-nut system [24]. In this model, the normal force $T_{n}$ varies with the rotation of the screw, and the resulting harmonic friction term can be described as follows:

(2) $$T_{ec}\left(\theta \right)=\beta sin\left(\theta -\theta_{0}\right),$$

where $T_{ec}$ and β are the eccentric friction torque and its amplitude, respectively. θ, and $\theta_{0}$ denote the current and initial angular positions of the screw, respectively, with $\theta =x\frac{2\pi }{L}$, where *x* is the linear displacement of the table and *L* is the lead of the screw.

The friction torque is related not only to velocity but also closely to feed acceleration due to the friction lag effect. To verify the impact of acceleration on friction, four sinusoidal trajectory experiments were conducted in the setup described in Section 3. The trajectory planning is outlined in Table 1, where *A* is the amplitude of the sinusoidal trajectory and ω represents the angular frequency.

**Figure 3:**
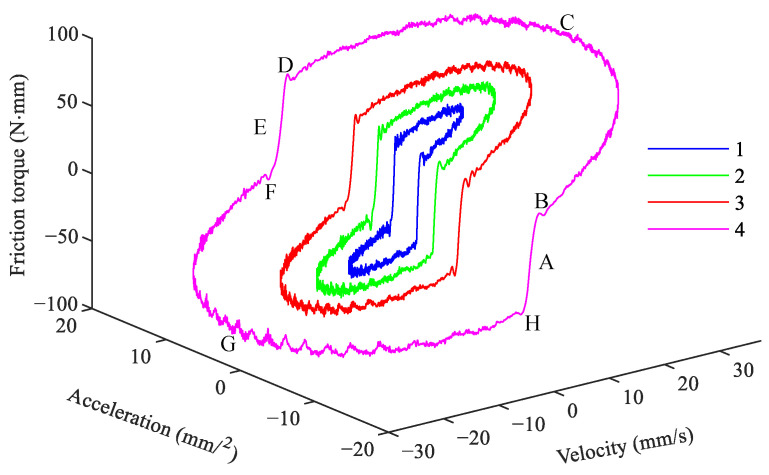
Three−dimensional curves of friction-velocity-acceleration.

**Figure 4:**
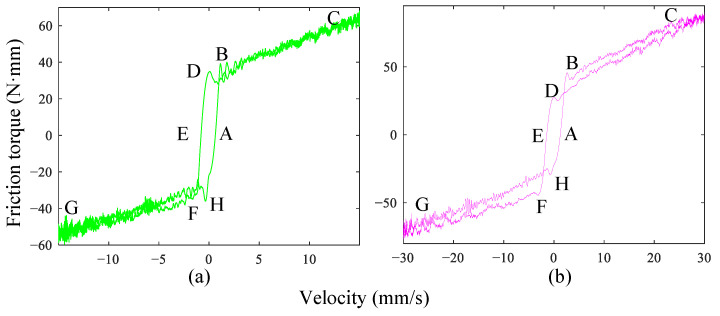
Curves of friction and speed. (**a**) the second experimental group. (**b**) the fourth experimental group.

The summary of the three-dimensional curves of friction-velocity-acceleration under the four experimental conditions is shown in Figure 3. As can be seen from the Figure 3, the acceleration of the four experiments is increasing. However, the effect of acceleration on friction cannot be well demonstrated. Figure 4a and Figure 4b are the friction-velocity curves for the second and fourth groups of experiments, respectively. While the acceleration a≠0 (*a* is motion acceleration), the conventional Stribeck friction model cannot describe the friction accurately.

As shown in Figure 4, the absolute value of friction in accelerating motion (B → C & F → G) is greater than that of decelerating motion (C → D & G → H) at the same speed, which is a typical characteristic of friction lag. There is almost no Stribeck effect in the partial fluid lubrication area when the system accelerates. However, at the time of deceleration, the Stribeck effect appeared in the partial fluid lubrication zone. In order to describe the friction lag caused by acceleration, this paper improves on the traditional Stribeck model based on the above friction characteristics:

(3) $$\left\{\begin{array}{c} T_{lag}\left(v,a\right)=\left[T_{c}+\left(T_{s}-T_{c}\right)e^{-{\left(vv_{0}^{-1}\right)}^{\delta }}S\left(av\right)\right]g\left(v\right)sgn\left(v\right)+\alpha v+T_{a}\left(a\right)\\ S\left(av\right)=\left\{\begin{array}{c} 1,sgn(av)>0\\ 0,sgn(av)<0 \end{array}\right.\\ g\left(v\right)=\frac{1-e^{-\lambda v}}{1+e^{-\lambda v}}\\ T_{a}\left(a\right)=sgn\left(a\right)\mu a, \end{array}\right.$$

where $T_{lag}$ is the friction lag caused by the acceleration. The meanings of *v*, $T_{c}$, $T_{s}$, $v_{0}$, and α are the same as those in Equation (1). μ and λ are the parameters that control the shape of the curve. δ is usually set to 2. The product of μ and *a* is used to describe the effect of acceleration on friction. The difference in the Stribeck effect is described by S(av) and g(v) in acceleration or deceleration.

From Equation (3), it can be seen that $T_{a}$ is a linear function of acceleration, so when the acceleration changes little, the formula can guarantee the accuracy of the prediction. However, in practical applications, many systems cannot avoid high-acceleration starting and stopping. At this time, the acceleration of the system changes greatly during the start-stop. The improved formula is as follows:

(4) $$T_{a}\left(a\right)=sgn\left(a\right)\frac{\xi_{0}}{1+\left|vv_{0}^{-1}\right|}\left(1-e^{-\left|a\xi_{1}^{-1}\right|}\right),$$

where $\xi_{0}$ and $\xi_{1}$ are all parameters to control the shape of the curve, $v_{0}$, *v*, *a* are Stribeck velocity, motion velocity, and motion acceleration, respectively.

From the above assumptions, a novel friction model is proposed, incorporating the Coulomb–Viscous friction, the friction lag component $T_{lag}$, and the eccentric friction term $T_{ec}$, and can be expressed as follows:

(5) $$\left\{\begin{array}{c} T_{d}\left(x,v,a\right)=T_{lag}+T_{ec}\\ T_{lag}\left(v,a\right)=\left[\eta_{0}+\left(\eta_{1}-\eta_{0}\right)e^{-{\left(v\eta_{2}^{-1}\right)}^{2}}S\left(av\right)\right]g\left(v\right)sgn\left(v\right)+\eta_{3}v+T_{a}\left(a\right)\\ S\left(av\right)=\left\{\begin{array}{c} 1,sgn(av)>0\\ 0,sgn(av)<0 \end{array}\right.\\ g\left(v\right)=\frac{1-e^{-\eta_{4}v}}{1+e^{-\eta_{4}v}}\\ T_{a}\left(a\right)=sgn\left(a\right)\frac{\eta_{5}}{1+\left|v\eta_{2}^{-1}\right|}\left(1-e^{-\left|a{\eta_{6}}^{-1}\right|}\right)\\ T_{ec}\left(x\right)=\eta_{7}sin\left(x\frac{2\pi }{L}-\eta_{8}\right), \end{array}\right.$$

where $\eta_{0}=T_{c}$, $\eta_{1}=T_{s}$, $\eta_{2}=v_{0}$, $\eta_{3}=\alpha$, $\eta_{4}=\lambda$, $\eta_{5}=\xi_{0}$, $\eta_{6}=\xi_{1}$, $\eta_{7}=\beta$ and $\eta_{8}=\theta_{0}$ in the new model. The model not only considers the Stribeck effect of friction, but also considers the nonlinear centrifugal friction caused by the eccentric screw nut and the lag effect of the friction. Therefore, the proposed model can describe the more precise friction behavior of the system. The estimation method of the proposed friction model will be explained in the next section.

## 3. Identification of Friction Models

### 3.1. Experimental Setup

**Figure 5:**
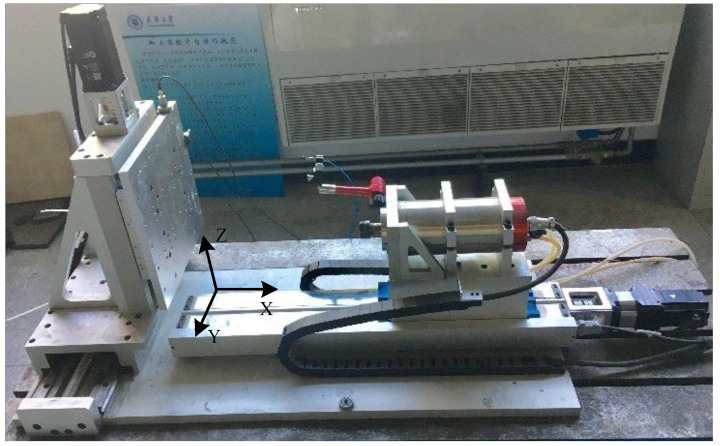
Three-axis machine tool.

The experimental system used in this study is a desktop three-axis machine tool. This paper focuses on the frictional properties of its feed drive axis, which is the X axis as shown in Figure 5. The feed drive axis was composed of a ball screw with a diameter of 16 mm and a lead of 5 mm connected to a gearbox with a reduction ratio of 5:1, and the gearbox is linked to a permanent magnet synchronous motor (400W, AC200V, YASKAWA, Shanghai, China) and a servo drive (SGDV, YASKAWA, Shanghai, China). The stroke of the feed drive was 330 mm. A real-time Programmable Multi-Axis Controller (PMAC) was used for motion control and data acquisition. In experiments, worktable motion data and friction torque data under different feed trajectories need to be obtained. The former includes worktable displacement *x* (mm), velocity *v* (mm/s), and acceleration *a* (mm/s²), obtained via a 1 μm resolution linear encoder and differentiation operations in the PMAC. The calculation methods for friction torque at constant and non-constant velocities are provided in Section 3.3 and Section 3.4, respectively. Furthermore, friction compensation program can be written using PMAC’s open servo algorithm.

### 3.2. Feed Drive Dynamics

**Figure 6:**
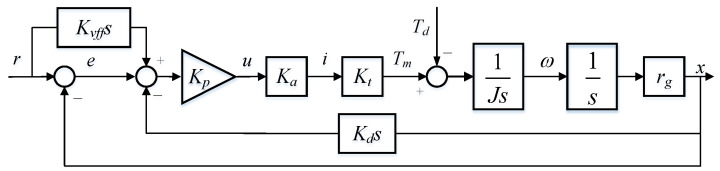
Linear dynamics of a feed drive.

The linear dynamics of the feed drive can be represented as shown in Figure 6. Here, $K_{p}$, $K_{d}$, and $K_{vff}$ are proportional gain, differential gain, and speed feed-forward gain of the control system, respectively. The desired position of the servo system is *r* (mm) and the actual position is *x* (mm), the difference between *r* and *x* is the trajectory following error *e* (mm). It is worth mentioning that the servo drive operates in torque mode, the torque loop frequency is much higher than the servo loop frequency. Consequently, the torque loop can be approximated as a proportional part with a gain coefficient of 1, meaning the driving torque applied to the motor is equal to the command torque. In this case, the motor driving torque $T_{m}$ can be calculated by multiplying the control law *u* by the amplifier gain $K_{a}$ (A/V) and the motor torque constant $K_{t}$ (N·m/A).

Furthermore, the motor driving torque $T_{m}$ overcomes the disturbance torque $T_{d}$, enabling the rotation of the screw shaft and movement of the worktable. *J* is the rotational inertia of the drive system. The lead screw gain $r_{g}$ is calculated as follows:

(6) $$r_{g}=\frac{L}{2\pi },$$

where *L* is the lead of the screw. This gain is used in a lead screw system to translate rotational motion to linear motion, and motor torque to driving torque. The parameter values of the feed drive system are given in Table 2, where the servo parameters $k_{p}$, $k_{d}$ and $k_{vff}$ are empirical parameters obtained by manual tuning, while the motor parameters $k_{a}$, $k_{t}$ and *J* are theoretical parameters obtained by the motor handbook.

### 3.3. Identification of Conventional Model

When the x-axis in Figure 5 is operated at a constant speed without machining, the disturbance is equal to the driving torque according to Newton’s second theorem and the main disturbance of the feed drive system is the friction torque. Therefore, the friction torque can be obtained by collecting the control law signal from the PMAC controller while the X-axis moves at a constant speed.

**Figure 7:**
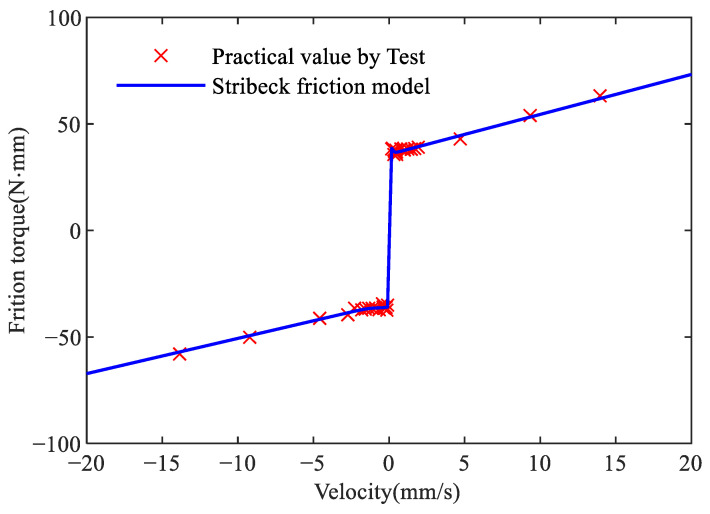
Tested friction and approximation by a conventional friction model.

Based on the estimation method in [20], actuate the feed drive at constant velocities of ±0.01, ±0.04, ±0.08, ±0.15, ±0.20, ±0.40, ±0.50, ±0.70, ±1.00, ±1.25, ±1.50, ±1.75, ±2.50, ±5.00, ±7.50, ±10.00, ±12.50, ±15.00 and ±17.50 mm/s. Figure 7 shows the tested and the fitted friction torque represented by Equation (1).

The Genetic toolbox algorithm in MATLAB is used to identify conventional friction model parameters. The population size and maximum genetic generations of the genetic algorithm are set to 200, and 10,000, respectively, to obtain the global optimal solution. The preset parameter search range can be established based on the physical meaning of the parameters. Data from forward and reverse feeding are used separately to identify the friction parameters in Equation (1): $T_{c+}$, $T_{c-}$, $T_{s+}$, $T_{s-}$, $v_{0+}$, $v_{0-}$, $\alpha_{+}$, and $\alpha_{-}$, where “+” indicates parameter in forward feeding and “−” indicates parameter in reverse feeding. The lower and upper bounds for these parameters are set to {10,−80,10,−80,−5,−5,−5,−5} and {80,10,80,10,5,5,5,5}, respectively. The fitness function *J* is described in Equation (7),

(7) $$J=\frac{1}{2}{\left(T-\hat{T}\right)}^{2},$$

where *T* and $\hat{T}$ are the collected and estimated friction torque. The obtained parameters of the conventional model are shown in Table 3.

### 3.4. Identification of the Proposed Friction Model

The identification method corresponding to the traditional Stribeck friction model requires numerous constant-speed experiments to draw the discrete points in Figure 7, which is cumbersome and time-consuming, and is prone to identification errors due to abnormal discrete points.

Therefore, this paper applies the steady-state error of the feed drive system to calculate the friction torque, facilitating rapid determination of the friction torque involved. The steady-state error of the system is defined as the steady-state component $e_{ss}\left(t\right)$ of the error signal e(t) as time approaches infinity. It is generally composed of two parts: one is caused by the desired input signal and the other is caused by the disturbance. The main source of disturbance in the drive system is friction torque.

Referring to Figure 6, the error transfer function of the desired input signal is as follows:

(8) $$\phi_{er}\left(s\right)=\frac{r}{e}=\frac{(K_{d}-K_{vff})s}{1+K_{d}s+\frac{J}{K_{p}K_{a}K_{t}K_{a}r_{g}}s^{2}},$$

where $K_{p}$, $K_{d}$, $K_{vff}$, $K_{a}$, $K_{t}$, *J*, and $r_{g}$ are proportional gain, differential gain, speed feed forward gain, current amplifier gain, motor torque constant, rotational inertia, and lead screw gain, respectively. If the condition $K_{d}=K_{vff}$ is satisfied, the steady-state error $e_{ss}\left(t\right)$ caused by the desired input signal is zero.

The error transfer function of the disturbance is as follows:

(9) $$\phi_{en}\left(s\right)=\frac{T_{d}}{e}=\frac{r_{g}}{K_{p}K_{a}K_{t}r_{g}+K_{p}K_{d}K_{a}K_{t}r_{g}s+Js^{2}}.$$

As is shown in Equation (9), when the disturbance is a step signal, the steady-state error $e_{ss}\left(t\right)$ caused by the disturbance is a certain value and when the disturbance is a ramp or uniform acceleration signal, the final value of the steady-state error $e_{ss}\left(t\right)$ is infinite. Under the influence of periodic variation in disturbance, the steady-state error $e_{ss}\left(t\right)$ of the system also presents periodic changes.

If the feed drive system satisfies the condition that $K_{d}=K_{vff}$, the relation between the steady-state error $e_{ss}\left(t\right)$ and the friction torque $T_{d}$ can be approximated by Equations (8) and (9) as follows:

(10) $$T_{d}=K_{p}K_{a}K_{t}e_{ss}\left(t\right).$$

The following sinusoidal reference is applied for the identification experiment of the proposed friction model:

(11) r=25sin(0.40t)−35(mm),t=[0,16]s.

**Figure 8:**
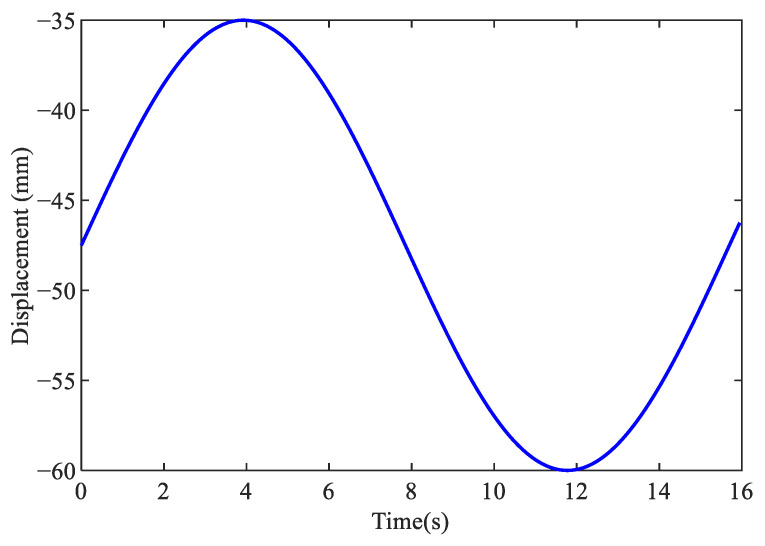
Reference for X-axis position.

**Figure 9:**
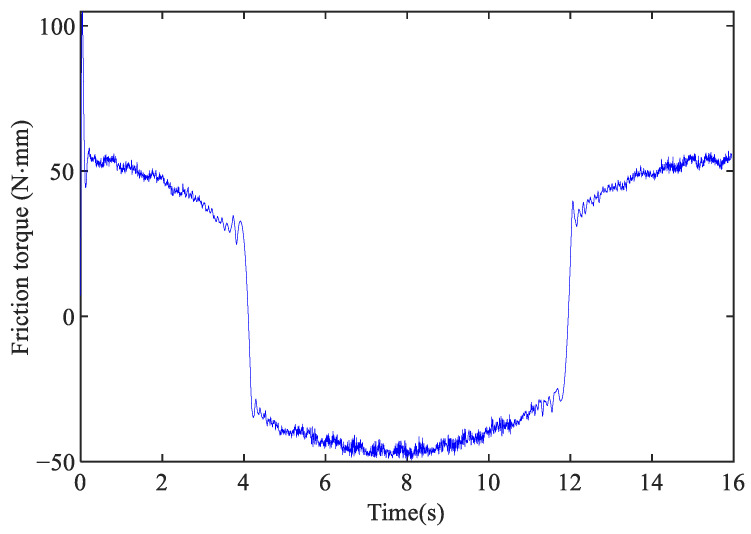
Frictional torque on X-axis.

The reference of the sinusoidal position and the friction torque calculated by Equation (10) are shown in Figure 8 and Figure 9, respectively. The genetic algorithm is used to identify the nominal Coulomb torque $\eta_{0}$, static friction $\eta_{1}$, Stribeck velocity $\eta_{2}$, nominal viscous coefficient $\eta_{3}$, and shape control coefficient $\eta_{4}$ as in [20]. Parameters $\eta_{0}$, $\eta_{1}$, $\eta_{2}$, $\eta_{3}$ are identified using the forward feed data and the reverse feed data, respectively, resulting in a total of nine parameters to be identified: $\eta_{0+}$, $\eta_{0-}$, $\eta_{1+}$, $\eta_{1-}$, $\eta_{2+}$, $\eta_{2-}$, $\eta_{3+}$, $\eta_{3-}$, $\eta_{4}$. The upper and lower bounds of the parameter changes preset in the genetic algorithm are set to {−80,−80,−80,−80,−5,−5,−5,−5,−5} and {80,80,80,80,5,5,5,5,5}, respectively, while the remaining parameters are the same as those in Section 3.3.

The estimated values are shown in Table 4. Comparing with the results in Table 3, it can be seen that the identification values of the Coulomb friction, static friction, Stribeck velocity, and nominal viscous coefficient are basically the same. However, the friction torque for the identification obtained from the steady-state error is more operative and applicable.

Utilize the uniform motion of the system to eliminate the effects of frictional lag, and obtain a more accurate centrifugal friction value. As the effect of friction lag is ignored, the eccentric friction value is calculated as follows:

(12) $$T_{ec}=T_{d}-\left[\eta_{0}+\left(\eta_{1}-\eta_{0}\right)e^{-{\left(vv_{0}^{-1}\right)}^{2}}\right]sgn\left(v\right)-\eta_{2}v.$$

The following ramp reference is applied for the identification of the eccentric friction value:

(13) r=−60+5t(mm),t=[0,12]s.

**Figure 10:**
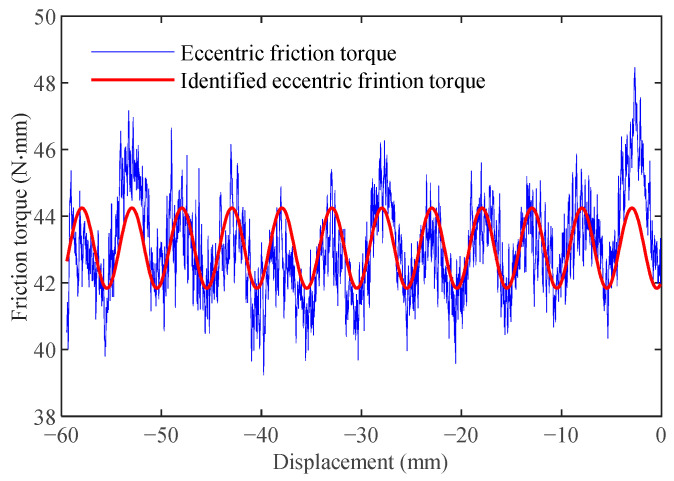
Eccentric friction.

The eccentric friction torque is shown in Figure 10, it consists of low-frequency sinusoidal curves and high-frequency noise. Since the tracking error signal used to estimate the friction torque in Equation (10) is sampled at 4000 Hz, noise components are inevitably present. These noise components are amplified by the coefficient $K_{p}K_{a}K_{t}$ and are reflected as burrs in Figure 10. Although there is noise, an obvious sinusoidal variation with a period of about 5 mm, which is close to the lead of the screw, can still be observed.

The genetic algorithm is used to identify parameters $\eta_{7}$ and $\eta_{8}$ of eccentric friction model in Equation (5). In the algorithm, the upper and lower bounds of the preset range are {−5,−5} and {5,5}, respectively, and the remaining parameters are the same as in Section 3.3. The result is shown as a red line in Figure 10. The estimated values of eccentric friction are shown in Table 4.

Continuing to use the sinusoidal command signal represented by Equation (11) for the identification of remaining friction lag parameters in the proposed model. After obtaining values based on the Stribeck model and centrifugal friction, the friction lag is calculated as follows:

(14) $$T_{lag}=T_{d}-\eta_{7}sin\left(x\frac{2\pi }{L}-\eta_{8}\right).$$

**Figure 11:**
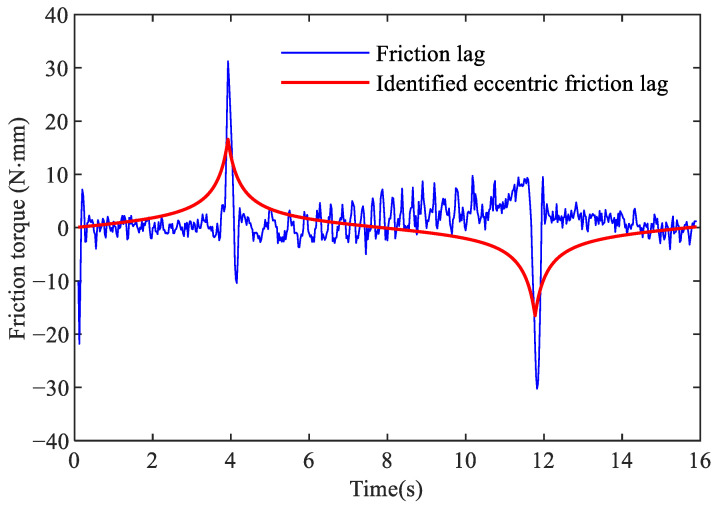
Friction lag.

The friction lag torque is shown in Figure 11. It can be seen that there are spikes on the left side and the middle of the curve. The spike on the left side is mainly caused by the sudden changes in acceleration of the motor in the start-stop phase, while the spikes in the middle are mainly caused by sudden changes in the speed direction when the motor commutates. Friction lag is closely related to the acceleration and the proposed friction lag model can describe the friction characteristics well.

The genetic algorithm is used to identify parameters of friction lag in Equation (5), while a sampling frequency of 40 Hz is used to mitigate the influence of high-frequency noise on identification accuracy. To capture the complex friction dynamic during velocity reversals, the fitness function in Equation (15) is utilized.

(15) $$J=\frac{1}{2}{\left(T-\hat{T}\right)}^{2}\left(1+ke^{-\left|v\right|}\right),$$

where *k* is the amplification factor and is set to 10 in this paper. The upper and lower bounds of parameters $\eta_{5}$ and $\eta_{6}$ in the genetic algorithm are set to {−1500,−1000} and {1500,1000}, while the other parameters remain consistent with those in Section 3.3. The result is shown as a red line in Figure 11. The estimated values of eccentric friction are shown in Table 4.

Finally, the friction torque for four different motion trajectories is predicted using the proposed friction model and the identified friction parameters. These trajectories are as follows:

- C1: Sinusoidal motion with 25 mm amplitude and 0.4 rad/s angular frequency;
- C2: Sinusoidal motion with 50 mm amplitude and 0.4 rad/s angular frequency;
- C3: Sinusoidal motion with 50 mm amplitude and 0.8 rad/s angular frequency;
- C4: S-Curve motion with 10 mm amplitude, 10 mm/s speed, and 100 ms acceleration time.

**Figure 12:**
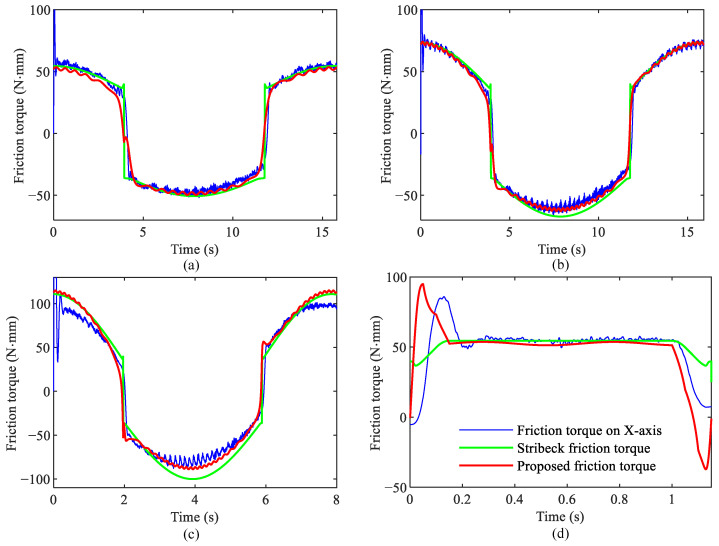
Comparison with conventional friction model. (**a**) Comparison for two friction models under trajectory C1; (**b**) under trajectory C2; (**c**) under trajectory C3; (**d**) under trajectory C4.

The predicted results are shown as the red line in Figure 12. In addition, the results predicted by the conventional Stribeck friction model are provided and shown as the green line for comparison purposes. From the identification results, the proposed friction model provides a better description of the friction dynamic. In Figure 12a,b, the predicted curves from the proposed friction model align closely with the actual friction torque. However, Figure 12c shows a slight deviation, which may be attributed to an overestimation of the identified viscous friction coefficient, resulting in prediction errors at high speeds. It can be seen from Figure 12d that the proposed model gives larger prediction results at the beginning and end, which means that the proposed model can give timely compensation during start-stop.

## 4. Friction Compensation and Experimental Results

The proposed nonlinear friction model is experimentally verified with the x-axis in Figure 5. Here, friction compensation is verified without machining a workpiece. We apply a user-written servo algorithm supported by the programmable multi-axis controller (PMAC) to compensate friction.

**Figure 13:**
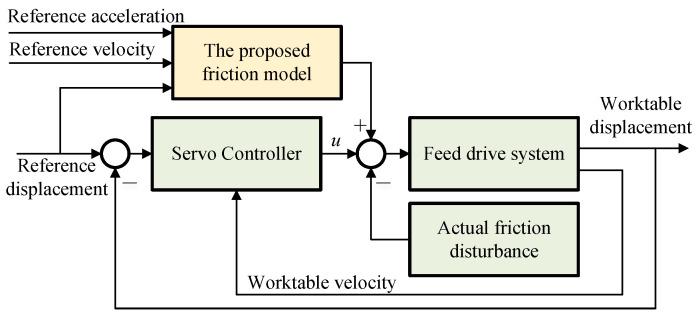
The friction feedforward compensation scheme.

The friction compensation scheme is illustrated in Figure 13. A friction feedforward compensation method is employed, which calculates the friction torque value using the reference worktable displacement, velocity, and acceleration, and then compensates it in the output of the original servo controller. This approach avoids the lag issue inherent in feedback compensation methods and provides excellent friction compensation performance.

**Figure 14:**
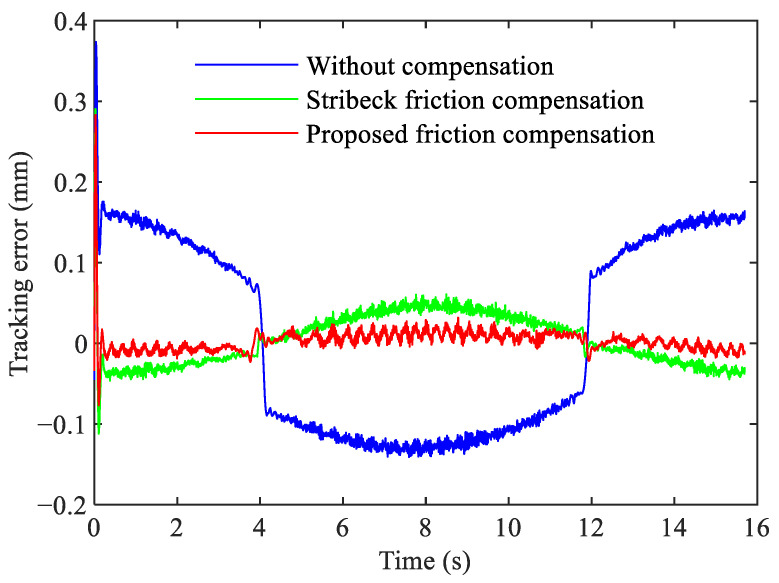
Comparison of friction compensation performance under different controller configurations. (blue line) without friction compensation. (green line) with Stribeck friction model compensation. (red line) with proposed friction model compensation.

The friction compensation performance was verified based on the tracking control results under the above four motion trajectories, while the same controller gains are used as $K_{p}=11,500$, $K_{d}=25,500$, and $K_{vff}=25,500$. The maximum absolute value and root mean square tracking errors are used as the performance indicators. The tracking errors of three different controller configurations under the C2 trajectory are compared, as shown in Figure 14.

It can be observed from Figure 14 that there is a large steady-state error without friction compensation and an obvious transient error occurs at startup. After compensating for friction, the steady-state error is greatly reduced. Compared to Stribeck friction compensation, the proposed friction compensation method results in a smaller steady-state error and ensures that the overall tracking error remains approximately zero.

**Figure 15:**
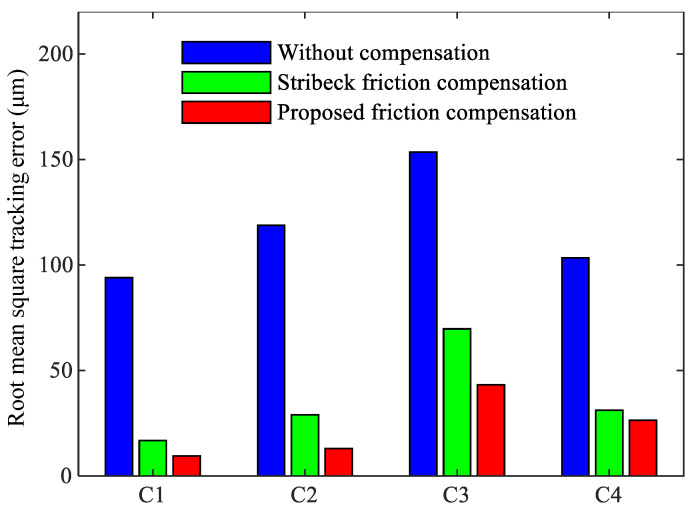
Root mean square tracking error results in sinusoidal reference.

**Figure 16:**
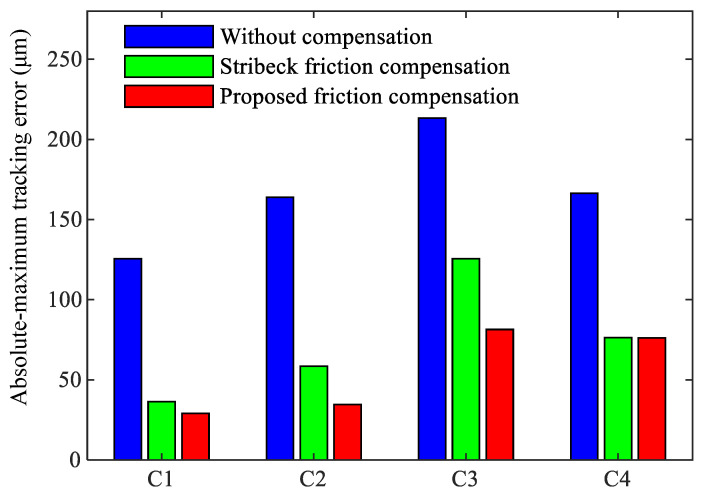
Absolute value of tracking error results in sinusoidal reference.

To further demonstrate the compensation effect, the maximum absolute tracking error and root mean square tracking error of the three controllers under four motion trajectories are compared in Figure 15 and Figure 16. Ten repeated tests were conducted for each motion trajectory, and the average values of the two indicators from these tests were calculated to ensure the reliability of the results.

Figure 15 and Figure 16 clearly show that for the four motion trajectories with varying amplitudes, speeds, and waveforms, the proposed friction compensation controller can achieve the best servo tracking performance. Compared to Stribeck friction compensation, the proposed friction compensation controller reduces the maximum absolute tracking error by 19.94%, 40.85%, 35.19%, and 0.23% for the four trajectories, respectively, and the root mean square tracking error is reduced by 35.71%, 39.46%, 25.03%, and 13.31%, respectively. Additionally, similar to the prediction results shown in Figure 12, for motion trajectory C3 with a higher feed-rate, the effectiveness of both Stribeck compensation and the proposed compensation is worse than that for C1 and C2. This may be due to the difference between the friction parameters identified under trajectory C1 and those of the actual system, which becomes more obvious in trajectory C3 with its higher feed speed.

## 5. Conclusions

Friction identification and compensation is an important artifice to improve the tracking accuracy of mechanical feed-drive systems. Although the classical friction models have an accurate description of the Stribeck effects, the characteristic of eccentric friction in a lead screw and friction lag caused by acceleration did not draw enough attention. This paper proposes a new nonlinear friction model that depends not only on velocity but also on position and acceleration to more accurately describe the characteristics of friction. We show how a small gap between a lead screw and nut leads to friction variations over one revolution of the screw and we also study the relationship between friction and acceleration. It is found that the absolute value of friction in accelerating motion is greater than that of decelerating motion at the same speed, whether the system is in an accelerating or decelerating motion, its friction torque is all around the Stribeck curve and forms a closed ring. Furthermore, there is almost no Stribeck effect in the partial fluid lubrication area when the system accelerates, but the Stribeck effect appears at the time of deceleration.

Experiments were conducted to compare the friction compensation performance between the proposed and the conventional friction models under four motion trajectories. The results show that the proposed method is more effective than the controller with the conventional friction compensation. On average, both the root mean square and maximum absolute tracking errors were significantly reduced after applying the proposed friction model compared to the compensation controller using the conventional friction model.

Simply updating the controller with the proposed nonlinear friction model allows for cost-effective improvement in the machining accuracy and positioning accuracy of machine tool drive systems. The current friction compensation only involves a single axis, as a result, the next research direction is to use the proposed new friction model to compensate for the contour error of multi-axis motion.

## Figures and Tables

**Table 1:** Experimental arrangement.

No.	*A* (mm)	ω (rad/s)
1	50	0.2
2	50	0.3
3	50	0.4
4	50	0.6

**Table 2:** Experimental arrangement.

Parameters	Value
$K_{p}$	11,500
$K_{d}$	12,500
$K_{vff}$	12,500
$K_{a}$ (A/V)	0.2335
$K_{t}$ (N·m/A)	0.544
*J* (kg·m^2^)	8.17 $\times \;10^{-5}$

**Table 3:** Estimated conventional Stribeck friction model parameters.

Parameters	Value	Parameters	Value
$T_{c+}$ (N·mm)	35.70	$v_{0+}$ (mm/s)	0.26
$T_{c-}$ (N·mm)	34.13	$v_{0-}$ (mm/s)	1.02
$T_{s+}$ (N·mm)	39.70	$\alpha_{+}$ (N/s)	1.88
$T_{s-}$ (N·mm)	35.81	$\alpha_{-}$ (N/s)	1.65

**Table 4:** Estimated nonlinear friction model parameters.

Parameters	Value	Parameters	Value	Parameters	Value
$\eta_{0+}$ (N·mm)	31.94	$\eta_{2+}$ (mm/s)	1.54	$\eta_{4}$	2.38
$\eta_{0-}$ (N·mm)	−34.48	$\eta_{2-}$ (mm/s)	−1.42	$\eta_{5}$ (N·mm)	939.95
$\eta_{1+}$ (N·mm)	27.14	$\eta_{3+}$ (N/s)	2.05	$\eta_{6}$ (mm/s^2^)	−201.239
$\eta_{1-}$ (N·mm)	9.98	$\eta_{3-}$ (N/s)	1.31	$\eta_{7}$ (N·mm)	1.20
				$\eta_{8}$ (rad)	1.03

## Data Availability

The original contributions presented in the study are included in the article. The authors will provide further support upon request.
